# A multiple antibiotic and serum resistant oligotrophic strain, *Klebsiella pneumoniae *MB45 having novel *dfrA30*, is sensitive to ZnO QDs

**DOI:** 10.1186/1476-0711-10-19

**Published:** 2011-05-19

**Authors:** Arvind Kumar, Soumynanda Chakraborti, Prachi Joshi, Pinak Chakrabarti, Ranadhir Chakraborty

**Affiliations:** 1 Omics Laboratory, Department of Biotechnology, University of North Bengal, Raja Rammohunpur, P.O. NBU, Siliguri 734 013, West Bengal, India; 2 Department of Biochemistry, Bose Institute, Kolkata 700 054, India; 3 National Physical Laboratory, New Delhi 110 012, India

## Abstract

**Background:**

The aim of this study was to describe a novel trimethoprim resistance gene cassette, designated *dfrA30*, within a class 1 integron in a facultatively oligotrophic, multiple antibiotic and human serum resistant test strain, MB45, in a population of oligotrophic bacteria isolated from the river Mahananda; and to test the efficiency of surface bound acetate on zinc oxide quantum dots (ZnO QDs) as bactericidal agent on MB45.

**Methods:**

Diluted Luria broth/Agar (10^-3^) media was used to cultivate the oligotrophic bacteria from water sample. Multiple antibiotic resistant bacteria were selected by employing replica plate method. A rapid assay was performed to determine the sensitivity/resistance of the test strain to human serum. Variable region of class 1 integron was cloned, sequenced and the expression of gene coding for antibiotic resistance was done in *Escherichia coli *JM 109. Identity of culture was determined by biochemical phenotyping and 16S rRNA gene sequence analyses. A phylogenetic tree was constructed based on representative trimethoprim resistance-mediating DfrA proteins retrieved from GenBank. Growth kinetic studies for the strain MB45 were performed in presence of varied concentrations of ZnO QDs.

**Results and conclusions:**

The facultatively oligotrophic strain, MB45, resistant to human serum and ten antibiotics trimethoprim, cotrimoxazole, ampicillin, gentamycin, netilmicin, tobramycin, chloramphenicol, cefotaxime, kanamycin and streptomycin, has been identified as a new strain of *Klebsiella pneumoniae*. A novel *dfr *gene, designated as *dfrA30*, found integrated in class 1 integron was responsible for resistance to trimethoprim in *Klebsiella pneumoniae *strain MB45. The growth of wild strain MB45 was 100% arrested at 500 mg/L concentration of ZnO QDs. To our knowledge this is the first report on application of ZnO quantum dots to kill multiple antibiotics and serum resistant *K. pneumoniae *strain.

## Background

*Klebsiellae *are ubiquitously present in nature and have been isolated from wide variety of habitats like-human body parts, animals, sewage, soils, vegetation, lakes, salt water, brackish water, fresh water and sachet water [1-3]. Generally, they are opportunistic pathogen for humans and other animals [1]. At present, nine validly published species have been reported for *Klebsiella *[4,5]. The genus comprises of non-motile, gram-negative, rod-shaped bacteria having a prominent polysaccharide capsule which encloses the total cell surface and renders resistance against several host defense mechanisms [6-8]. Strains of *K. pneumoniae *conferring resistance to an extended spectrum β-lactams, carbapenems, cephalosporins, aminoglycosides, flouroquinolones together with the trimethoprim (TMP) and cotrimoxazole and other antibiotics, have been isolated from different clinical setup [9-13]. Trimethoprim is used as primary drug in the prophylaxis and treatment of both urinary and respiratory tract infections [14]. Resistance to trimethoprim is caused by modifications in the target enzyme dihydrofolate reductase, encoded by *dfr *genes located either on plasmid or chromosome [15-20]. Different *dfrA *genes (> 25) conferring resistance to trimethoprim have been reported and 15 of them were integron-borne [15,21-23]. Integrons are genetic elements that contain determinants of a site-specific recombination system by which they can excise or integrate gene cassettes (mobile element), usually antibiotic resistance genes encoding antimicrobial resistance [24,25]. Presence of class 1 integrons is documented in both eutrophic and oligotrophic bacteria isolated from Indian rivers of northern West Bengal [23,26]. There were number of attempts to cultivate oligotrophic bacteria using different diluted media [27,28] including R2A media [29]. Recently, diluted Luria broth (LB) was used to cultivate oligotrophic bacteria from environmental sample [23,30]. There are reports on oligotrophic bacteria isolated from clinical materials [28] and quite a good number of oligotrophic bacteria in river waters exhibit antibiotic resistance [23,30]. Hence, oligotrophs could be a potential reservoir of antibiotic resistance genes that can be acquired by pathogens through diverse gene transfer mechanisms. Due to their potential clinical importance, oligotrophic bacteria merit attention. The prevalence of multiple antibiotic resistant bacteria among pathogens and by-standers (normally not pathogenic but becomes virulent under immunosuppressive conditions) poses a severe threat to public health worldwide. It is difficult to eradicate antibiotic resistant pathogenic bacteria which can survive in low nutrient condition for a long period of time (oligotrophs). As mortality and morbidity rate due to infection by multiple-antibiotic-resistant bacteria is on rise, novel therapeutic strategies are being devised to combat this problem. Metal oxide nanoparticles have shown antimicrobial property (31). Among different metal oxide nanoparticles, ZnO being non-toxic is popular due to its biocompatibility. Testing of ZnO Quantum dots as antimicrobials is mainly done on gram-negative antibiotic-sensitive strain of *E. coli *[31,32].

In this study, we have demonstrated the efficacy of ZnO Quantum dots to inhibit growth of an oligotrophic, multiple antibiotic and serum resistant strain of *K. pneumoniae *MB45 isolated from river Mahananda of Siliguri, West Bengal, India. We have also characterized and expressed a novel class 1 integron borne trimethoprim resistant *dfrA *gene, designated *dfrA30 *from the said strain.

## Methods

### Sampling, Isolation and selection of the Test strain, and preparation of Antibiogram

The strain was isolated from river water sample. Sampling, isolation and identification of oligotrophic bacteria were done according to the methods described earlier [23]. Replica plating method was employed for determining the antibiogram of the isolate designated as MB45 [23]. Strain, MB45, resisting high level of trimethoprim (>1500 mg/L) was selected for this study. The susceptibility of MB45 tested as described previously [23]. Criteria for susceptibility followed the EUCAST guidelines http://www.eucast.org/clinical_breakpoints/. Susceptibility to the antibiotics absent in EUCAST breakpoints table (v 1.1 2010-04-27), were interpreted according to previously described criteria [23]. Strain was maintained by bi-weekly transfer to R2A agar slants (HiMedia, India) and stored at -20°C in R2A broth amended with glycerol (20% v/v).

### Phenotypic and Phylogenetic characterization of MB45

All phenotypic and biochemical test were performed following methodology described earlier [23] at 37°C. Growths at different temperatures were tested in LB at 7, 10, 15, 25, 30, 37, and 45 ± 1°C. DNA for amplification of 16S rRNA gene was extracted from cells (grown in LB for 4 hr at 37°C) by boiling lysis method. A loop full bacterial culture (24 h old) was inoculated in 100 mL flask containing 10 mL LB and incubated for 4 hr at 37°C without agitation. The cells (0.5 mL) were harvested by centrifuging at 6000 rpm for 10 minute at 4°C. The pellet was suspended in 200 μL sterile distilled water and put in a microwave oven for 1.5 min at 800 watt (LG model No MS-194W). Lysate was cooled at room temperature and centrifuged at 8000 rpm for 2 min to remove cell debris. 2 μL of the resulting supernatant was used as template DNA in 25 μL of PCR mix. Amplification, cloning and sequencing were done according to the previously described method [23].

### Viability and Growth of MB45 in diluted (10^-3^) Luria-broth

Inoculum was prepared by transferring a single colony of 24 h old culture of MB45 into 10 mL sterile LB (pH 7.0) in 100 mL Erlenmeyer flask. The inoculated medium was incubated at 37°C for 4 h without agitation. The culture was harvested by centrifuging at 8 000 rpm for 5 min at 4°C and washed twice with sterile saline (0.5% NaCl) water to remove traces of media. The washed pellet was finally suspended in 3 mL sterile saline water. Aliquots of 1.0 mL of concentrated (1 × 10^8 ^cells/mL) cell suspension (s) were added to 25 mL of diluted (10^-3^) LB in 250 mL Erlenmeyer flask. The flask was kept at 37°C (without shaking) throughout the period of investigation. Survivability of MB45 cells in 10^-3 ^LB was assessed through dilution-plating of pure culture aliquots at different time intervals on fresh LA plates.

### Serum bactericidal assay

Serum bactericidal assay was basically performed by the methods described by Sharma et al. [33].

### Growth kinetics of MB45 in presence of varied concentrations of ZnO QDs

ZnO QDs with surface adsorbed acetate ions (particle size 3-5 nm) was synthesized and characterized by methods described earlier [31]. Inoculum, for testing ZnO QDs efficacy, was prepared as described above. Aliquots of 10 μL of concentrated (9 × 10^7 ^cells) cell suspension(s) were added to 3.0 mL volume(s) of LB in 10 mL glass test tube(s) amended with different concentrations of ZnO QDs. The tube containing all the ingredients except ZnO QDs was taken as positive control. LB containing ZnO QDs but lacking cells were taken as negative control. The tubes were kept at 37°C with continuous agitation at 200 rpm throughout the period of investigation. Each test was performed in triplicate. The optical density was measured in spectrophotometer (Model-302, Electronic India) at 600 nm at different time intervals. Growth rate constant (μ) and mean generation time (g) of MB45 in absence and presence of different concentrations of ZnO QDs were calculated.

### Phylogenetic affiliation of MB45

Phylogenetic analysis of the 16S rRNA gene sequences of MB45 and all the known species of genus *Klebsiella *were conducted in the software package MEGA4 [34]. Multiple alignments of sequences were done with CLUSTAL W [35]. Distances were calculated according to the Jukes-Cantor. Phylogenetic analyses were performed using two tree-making algorithms: the NJ (neighbor-joining) [36] and MP (maximum parsimony) [37] methods to ensure consistency of the clusters formed (data not shown). Tree topology was evaluated by the bootstrap re-sampling method of Felsenstein [38] based on 1000 replication.

### Detection of class 1 integron, cloning, sequencing, and expression of variable region

Whole cell DNA was extracted as described above. Variable region of the class 1 integron was amplified using two sets of primer pair, 5" CS and 3" CS; and Int_2_F and 3" CS described previously [39,40]. Cloning and sequencing were done according to the method described previously [23]. For determination of resistance coded by *dfrA30 *gene, the amplicon was cloned in pGEM-T easy vector system-II (Promega, Madison, USA) and then transformed into *E. coli *JM109. Clones containing insert (CS-PCR product) in proper orientation were selected on LA plates containing IPTG and X-gal (for Blue-White screening) and trimethoprim (5 mg/L). One of the clones (pAK45) appeared on trimethoprim amended LA plate was purified, stored and used for testing the maximum tolerance of trimethoprim. Plasmidless *E. coli *JM109 was used as control.

### Sequence analysis and phylogenetic position of the *dfrA30 *gene

Each DfrA protein sequences were obtained from GenBank database (NCBI, http://www.ncbi.nlm.nih.gov/). The phylogenetic analysis at the amino acid level of the DfrA30 (translated *dfrA30 *gene) was done by constructing a phylogenetic tree based on one representative for each trimethoprim-resistance-mediating DfrA proteins. The multiple alignments were made using the program CLUSTAL W [35]. The phylogenetic tree was constructed using NJ tree-making algorithm. The consistency of the clusters formed was also validated by methods MP [37] and UPGMA (Unweighted Pair Group Method with Arithmetic mean) [41] (data not shown). Sequence alignment and phylogenetic analysis of the Dfr protein sequences were accomplished in MEGA4 [34].

### Sequence alignment and the effect of mutation in DfrA30

To identify the residues responsible for trimethoprim-resistance in the DfrA30, multiple sequence alignment of DfrA proteins [DfrA30, Ac. No. AM997279; DfrA5, Ac. No. AJ419169; and wild type (WT, trimethoprim-sensitive) Dfr, Ac. No. J01609 ] was carried out using CLUSTAL W [35]. Due to the non availability of the trimethoprim-bound structure from *E. coli *in the Protein Data Bank (PDB) [42], sequence alignment was also performed between different trimethoprim-bound Dfr available in PDB. The protein from the *Mycobacterium avium *(PDB ID: 2W3V) gave the highest score and was used for the analysis of ligand binding. Pymol http://www.pymol.org was used for molecular visualization.

### Nucleotide accession number

The EMBL accession numbers for the 16S rRNA gene and *dfrA30 *gene are respectively FR677021 and AM997279.

## Results

### Phenotypic and phylogenetic characterization of MB45

**Figure 1:**
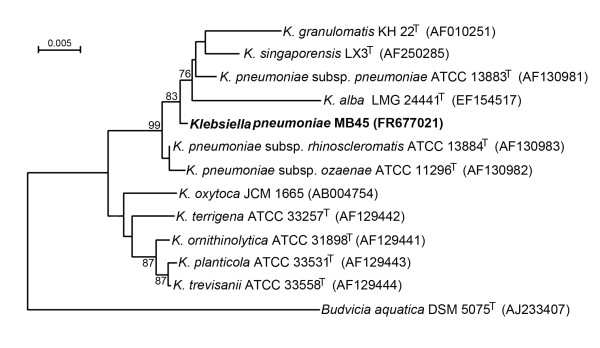
**N-J tree based on 16S rRNA gene sequences showing the position of strain MB45 (Bold face) within the members of genus *Klebsiella***. *Budvicia aquatica *DSM 5075^T ^(AJ233407) used as an outgroup. Bootstrap values (>70%), expressed as a percentage of 1000 replications, are given at branching points. EMBL/GenBank accession numbers are given in parentheses. Bar, 0.005 substitutions per nucleotide position.

Cells of the strain MB45 were rod shaped, capsulated, gram negative, aerobic (facultative anaerobic) and non-motile. On Luria agar, colonies were circular, convex, translucent, mucoid, sticky and off-white in colour with diameters of 2.0-3.0 mm after 3 days at 37°C. Differential phenotypic and biochemical characteristics of the strain MB45 and nearest strains are given in Table 1. Antimicrobial susceptibility testing (recommended by EUCAST for Enterobacteriaceae) showed that the strain MB45 was resistant to trimethoprim (S≤/R >, 2/4 mg/L), cotrimoxazole (S≤/R > 2/4 mg/L), ampicillin (R > 8 mg/L), gentamycin (S≤/R > 2/4 mg/L), netilmicin (S≤/R > 2/4 mg/L), tobramycin (S≤/R > 2/4 mg/L), chloramphenicol (S≤/R > 8/8 mg/L) and cefotaxime (S≤/R > 1/4 mg/L) and antibiotics, absent in EUCAST- kanamycin (R≥ 5 mg/L) and streptomycin (R≥ 2.5 mg/L). Nearly complete 16S rRNA gene sequence (1503 bp) was amplified, cloned and sequenced. The 16S rRNA gene sequence similarities with phylogenetic neighbours were in the range 97.5-99.6%. The closest species to the strain MB45 was *K. pneumoniae *subsp. *rhinoscleromatis *ATCC 13884^T ^(99.6% sequence similarity). In the NJ tree constructed with 16S rRNA gene sequence of *K. pneumoniae *MB45 formed a tight clade with a bootstrap support of 83% with the cluster comprising the four *Klebsiella *strains named as *K. pneumoniae *subsp. *pneumoniae*, *K. granulomatis*, *K. singaporensis *and *K. alba *(Figure 1). Similar output was obtained from MP clustering (data not shown).

**Table 1:** Differential biochemical properties of *K. pneumoniae *MB45 from other *Klebsiella *species

*Characteristics*	*MB45*	*1*	*2*	*3*
Gas from Lactose at 44 ± 1°C	-	+	+	+
Methyl red	-	-	+	+
Voges Proskauer	+	+	-	-
Urease	-	+	-	-
ONPG#	+	+	-	+
Malonate utilization	+	+	+	-
Lysine decarboxylate	+	+	-	d
Ornithine decarboxylase	+	-	-	-

Strains: MB45; 1, *K. pneumoniae *subsp. *pneumoniae*; 2, *K. pneumoniae *subsp. *rhinoscleromatis*; 3, *K. pneumoniae *subsp. *ozaenae*

Abbreviations: +, positive; -, negative; d, 11-89% positive; #, *o*-nitrophenyl-β-D-galactopyranoside

Data from this study and previous studies [4,69,70]

### Growth of the strain MB45 in diluted (10^-3^) Luria broth

**Figure 2:**
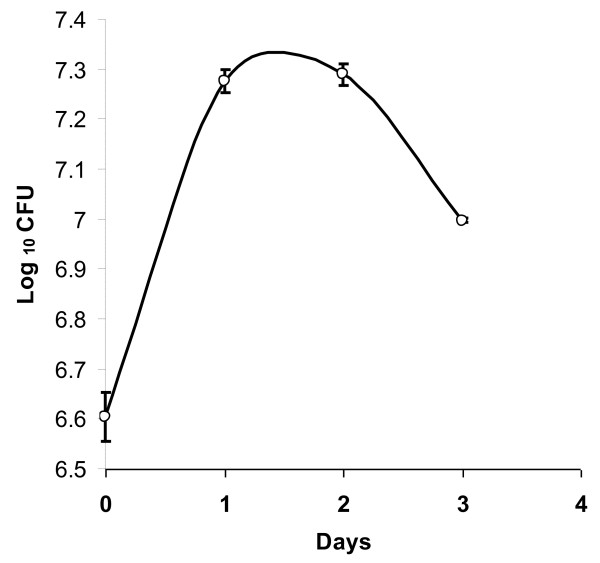
**Viability and growth in diluted (10**^**-3**^**) Luria broth**. Bars shows standard error.

The growth of MB45 cells were observed in diluted (10^-3^) LB lacking any extra supplementation of growth factor (Figure 2). An increase of 4.6 times of the initial cell number was noted in 2 days. The ability of MB45 to survive (without reduction in viable cell number since inoculation) and grow in a low nutrient medium explains the oligotrophic nature of the strain.

### Serum bactericidal activity

The serum reactivity against the strain MB45 was determined by the rapid assay. MB45 could change the colour in NHS and HIS tubes at 5 h and therefore inferred as serum-resistant. The control isolate, *E. coli *K12 could not change the colour of NHS tube even at 8 h, but turned HIS tube yellow at 5 h, was regarded as serum-sensitive.

### Effect of ZnO QDs on the growth of MB45

**Figure 3:**
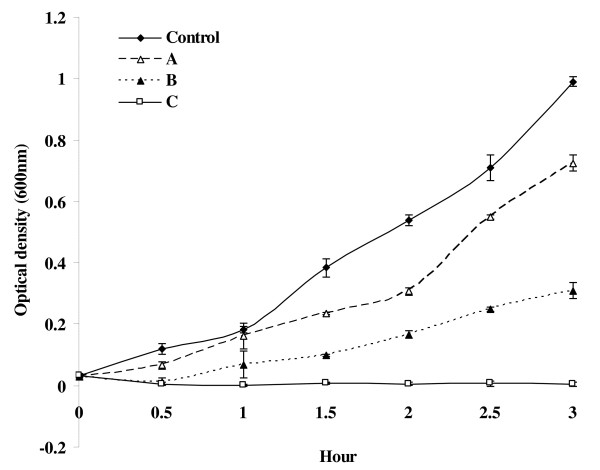
**Effect of ZnO QDs on the growth of MB45**. Cultures were setup with an initial cell number 9 × 10^6 ^cells in Luria broth amended with different concentration [solid rhombus (♦), Control 0.0 mg/L; open triangle (∆), A 200 mg/L; filled triangle (▲), B 400 mg/L; open rectangle (□), C 500 mg/L] of colloidal solution of ZnO QDs. Cultures were incubated at 37°C with constant shaking at 200 rpm under normal laboratory condition. Bars shows standard error.

The growth curves of MB45 in presence of different concentrations of ZnO QDs were shown in Figure 3. The bacterial growth rate (in comparison to the control, without QDs) was found to get slower with increasing concentrations of ZnO QDs. The growth rate constant and mean generation time in LB without ZnO QDs (μ = 0.019 min^-1^, g = 36.5 min) was significantly affected on addition of ZnO QDs (μ = 0.013 min^-1^; g = 53.3 min; at 400 mg/L). Growth was completely arrested at 500 mg/L QDs.

### Description of class 1 integron and phylogenetic position of *dfrA30*

**Figure 4:**
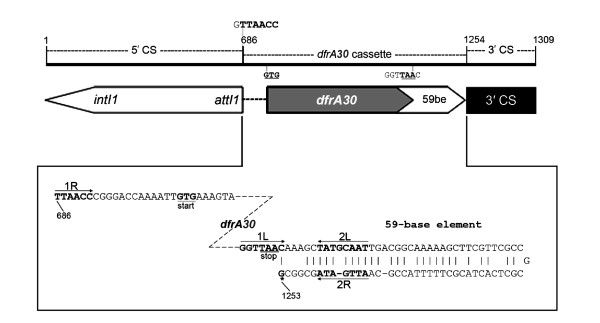
**Schematic representation of the Int**_**2**_**F-3'CS amplicon of the strain MB45**. CS, conserved segment; *int*I1, integrase gene; *att*I1, attachment site; *dfr*, dihydrofolate reductase; be, base element. Black thick bar shows the distribution of integron features on amplified product. The translation start (GTG) and stop (TAA) codon are in underlined bold face. In the 59 be, the putative integrase binding sites 1L, 2L, 1R and 2R are indicated by arrows. Termination of 59 be is indicated by star (*). Numbers correspond to sequence positions in EMBL Ac. No. AM997279.

**Figure 5:**
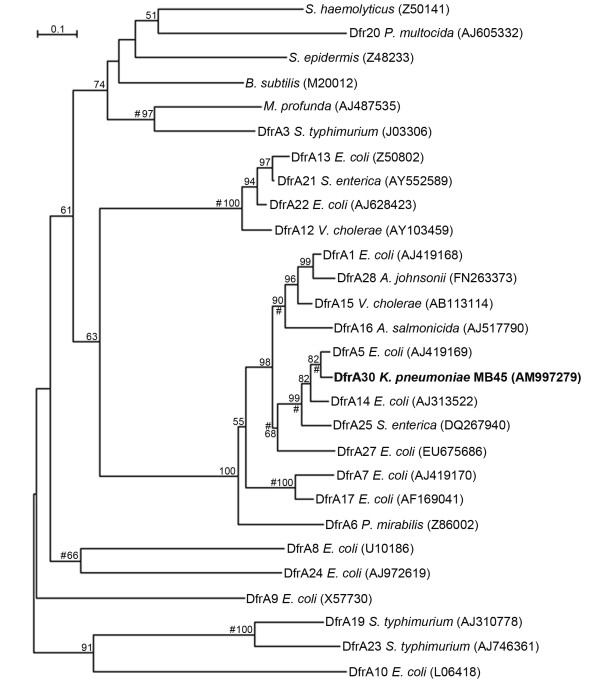
**Phylogenetic tree showing the position of DfrA30 (Bold face) within the DfrA proteins involved in trimethoprim resistance**. The number (>50%) at each major branch point refers to the percentage of times that a particular node was found in 1,000 bootstrap replications. Source and the GenBank/EMBL/DDBJ accession (in parentheses) numbers are given for each DfrA protein. DfrB proteins, which differ distinctly from DfrA proteins in size and structure, are not included in this tree. Common clusters obtained from NJ, MP and UPGMA tree are represented by hash (#).

The Int_2_F and 3" CS primer pair yielded ~1.3 kb amplicon from the strain MB45, comprising a single gene cassette. The gene cassette contained a 471 bp long ORF with 93% identity at the amino acid level to the DfrA5 (Ac. No. AJ419169) of *E. coli*. The deduced DfrA30 protein sequence consists of 157 amino acids and thus was in the same size range as of DfrA5 (157 amino acids). Amino acid sequence comparison (identity) between the DfrA30 and the known TMP-resistant Dfr proteins ranges from 15.1-93%. Maximum identity (93% and 88.5%) of DfrA30 was with the Dfr proteins of *E. coli *(DfrA5, Ac. No. AJ419169 and DfrA14, Ac. No. AJ313522 respectively) and least (15.1%) with the DfrA23 of *S. typhimurium *(Ac. No. AJ746361). The DfrA30 was 33.1% identical to the chromosomal Dfr protein (encoded by *folA*) of *E. coli *K12 (Ac No. J01609). The 5" conserved sequence which terminates at the *attI*1 core site G/TTA, which marks the point of insertion and beginning of first gene cassette (EMBL nucleotide sequence position, 686; Ac. No. AM997279) was identical to those of class 1 integrons. The core site for the site-specific insertion, GTTAACC, was located at position 685-691. The ORF began with the initiation codon GTG at positions 705 to 707 and terminated with the stop codon TAA (within the inverse core site) at positions 1176 to 1178. Downstream to the 3" end of the *dfrA30 *gene, an 81 bp long structure, recognized as an *attC *site (59 base element) began with the sequence GGTTAAC (1L, inverse core site at position 1173-1179) and terminated with the core sequence GTTAGAT (EMBL nucleotide sequence position, 1253). Integrase binding domains 2L (TATGCAAT; position, 1185-1192) and 2R (ATTGATA; position, 1241-1247) within the 59 base element were also identified (Figure 4). Novel DfrA (DfrA30) from MB45 branched deeply with DfrA5 of *E coli *in the NJ phylogenetic tree (Figure 5).

The clone, pAK45, conferred resistance to trimethoprim (MIC, 1000 mg/L) and ampicillin (selection marker). The level of trimethoprim-resistance of the wild strain MB45 was noted >1500 mg/L. The MIC of the control strain (plasmidless JM109) was restricted to 5 mg/L for trimethoprim.

Since the identity of translated *dfrA *gene of MB45 was 93% with its nearest variant *dfrA5 *gene (and this particular sequence deposited in the GenBank was annotated with the most similar features from the Feature database (FDB) as *dfrA30 *[43]), this gene was predicted to be a new *dfrA *gene and was named *dfrA30 *following guidelines for naming new trimethoprim resistance genes [15,44].

### Sequence alignment and Mutation study of DfrA30

**Figure 6:**
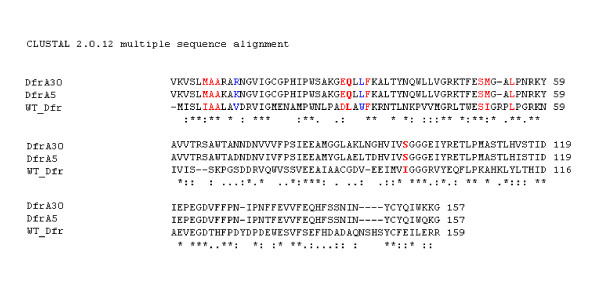
**Sequence alignment of DfrA30 with DfrA5 and sensitive WT-Dfr protein**. The residues which form the binding site [43] are shown in red, and the positions of mutations from the wild type (WT) protein are shown in red bold letters. Residues (beyond the active site) which are known to play an important role in trimethoprim binding [44], and which have been mutated are marked in blue.

**Figure 7:**
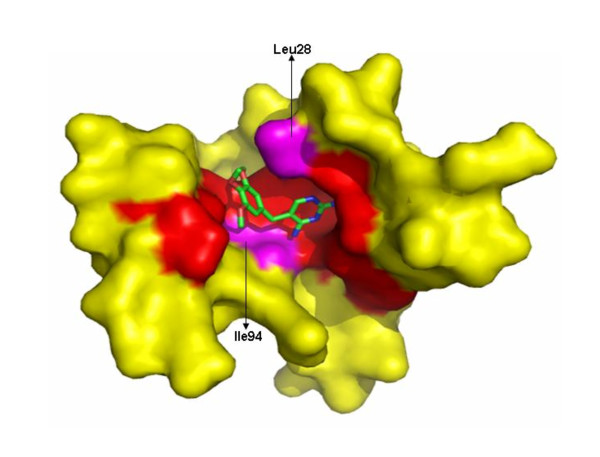
**Surface representation of TMP binding site on DFR (PDB ID: **2W3V**)**. Residues which form the active pocket are shown in red, and the two residues that are mutated in DfrA30 are in magenta; the remaining residues are in yellow. TMP is shown in stick model.

Multiple sequence alignment of DfrA30, DfrA5 and TMP-sensitive wild type (WT) Dfr protein (encoded by *folA *gene, Ac. No. J01609)] was done. Residues constituting the binding site for TMP [45], positions of the mutated amino acids in the active site of DfrA30 with respect to the wild type protein and also the non active site residues that are known to play a vital role in TMP binding were identified (Figure 6) [46]. The 3-dimensional structure of Dfr (PDB ID: 2W3V) depicting the active site pocket including the two important residues (positions 28 and 94) for TMP binding was analysed (Figure 7). The sequence alignment between wild type (WT) and *Mycobacterium avium *Dfr (PDB ID: 2W3V) (which was used to visualize the ligand binding) is available as Additional file 1: Figure S1.

## Discussion

Recent studies have shown that the frequency of antibiotic resistance has been the second highest in the genus *Klebsiella *especially in *K. pneumoniae *(next to *E. coli*) within the family *Enterobacteriaceae*; and the rate of occurrence has been noted to be higher in isolates from developing countries than the developed countries [12,47-49]. The incidence rates of trimethoprim resistance in *Klebsiella *spp. and *E. coli *in particular have been alarming [12,50-52] in the context of an earlier surveillance study (1987-88) on community isolates revealing an increase in trimethoprim resistance from 15.2% to 24% in *Klebsiella/Enterobacter *spp [17]. The most frequent mechanism of bacterial trimethoprim-resistance is the production of an additional trimethoprim-resistant Dfr, regularly found on mobile genetic elements (plasmids, transposons, gene cassettes) [14,17]. Other mechanisms of bacterial resistance to trimethoprim that have been described are impermeability (found in isolates of *Serratia*, *Enterobacter*, *Klebsiella*, *Pseudomonas*, and *Clostridium*) and mutational changes in the thymidylate synthase gene [14,17,53]. Besides antibiotic resistance, incidence of serum resistance in clinical isolates of pathogenic bacteria is an additional threat. There appears to be a strong correlation between serum resistance and the ability of a variety of gram-negative bacteria to invade and survive in the human blood stream [54]. Earlier authors have developed a simple and rapid assay for determination of serum bactericidal activity using more than hundred clinical isolates of *K. pneumoniae *and have found that 50% were resistant to 20% normal human serum [33]. Nosocomial septicemia due to extended spectrum β-lactamase producing *K. pneumoniae *and *E. coli *are a therapeutic challenge due to resistance [9]. Recently, it was shown that treatment without resistance selection at the infection site with fluoroquinolone treatment can be linked to colonization of the digestive tract by *K. pneumoniae *(targeted pulmonary bacteria), followed by the emergence of resistance [8]. Complete sequence of *K. pneumoniae *multidrug resistance plasmid pKP048 has revealed the presence of several important resistance genes, such as *bla *(KPC-2), *bla *(DHA-1), *qnrB4*, and *armA*, which confer resistance to carbapenems, cephalosporins, fluoroquinolones, and aminoglycosides, respectively [10]. In the present study, the test strain, MB45, isolated from river Mahananda at Siliguri, India, is resistant to serum as well as to antibiotics trimethoprim, cotrimoxazole, ampicillin, gentamycin, netilmicin, tobramycin, chloramphenicol, cefotaxime, kanamycin and streptomycin and could survive in low nutrient condition (oligotrophic).

Characterization of integron-borne cassette arrays in *K. pneumonia *strains from China revealed a predominance of *dfr *and *aadA *cassettes that confer resistance to trimethoprim and aminoglycosides respectively [55]. However, the distribution of *dfr *genes in *K. pneumoniae *was not known until recently a study on trimethoprim resistance in 54 trimethoprim resistant *K. pneumoniae *isolates have revealed the presence of *dfrA1*, *dfrA5*, *dfrA7*, *dfrA8*, *dfrA12*, *dfrA14*, *dfrA17 *and class 1 and 2 integrons; *dfrA1 *and *dfrA17 *being most prevalent and rarest respectively [48]. More than 25 different TMP-resistance-mediating *dfr *genes isolated from different bacteria, subdivided into two major types, 1 and 2 (referred as *dfrA *and *dfrB*), have been observed till date [56,57]. A novel trimethoprim resistance gene is claimed when the translated Dfr encoded by the gene has <95% identity at the amino acid level compared with known Dfr proteins [15]. Since the degrees of identity between the DfrA30 and the protein sequences of other Dfr(s) ranged between 15.1 and 93%, thus placing DfrA5 and DfrA30 in an indisputable monophyletic group in the phylogenetic analysis (Figure 5).

The strain MB45 showed high level of resistance to TMP (>1500 mg/L). Generally, a single mutation in the active site is enough for TMP-resistance in Dfr, though multiple mutations are common in clinically isolated species. The mutations in the active site residues reduce the binding affinity of the enzyme for the drug. Matthews and co-workers have identified the residues that constitute the TMP-binding site in *E. coli *Dfr(Figure 6) [45]. Additionally, the mutations in the active site that lead to TMP resistance in *E. coli *have been enunciated [46]. The mutations in DfrA30 are of the same type as those in DfrA5. In particular, two changes, glutamine for leucine at residue 28 and isoleucine to serine at 94, would change the hydrophobic/polar nature at the two opposite sides of the TMP site (Figure 7), thus possibly weakening the binding. Other mutations (V10, W30 and I94) beyond the active site have also been identified in clinically isolated TMP-resistant genes [46]. Some of these are also found in DfrA30 (mutations V10K and W31L) (Figure 6).

The percent occurrence of integron-positive isolates from clinical samples was much higher compared to environmental samples including fish farms [58-62], irrigation water sources [63] and other aquatic environments [23,26,64-66]. Except one recent study [23], in all other studies the incidence of class 1 integrons was observed for copiotrophic isolates that grow on rich nutrient medium. The test strain, MB45, is a multiple-antibiotic resistant oligotrophic bacteria recovered on 0.001X LA from river Mahananda. The facultatively oligotrophic strain used in this study was characterized as *K. pneumoniae *MB45 (ascertained from phenotype as well as from 16S rRNA phylogeny) (Figure 1). Viability assay and growth assessment of *K. pneumoniae *MB45 cell concentrate in 0.001X Luria broth for more than 72 h by taking viable cell count of the cell suspension on 1X LA at different times (Figure 2) demonstrated its ability to adapt both oligotrophic (ability to survive and grow in extremely poor nutrient conditions) as well as copiotrophic (ability to form colonies in a rich medium) conditions of growth. Such facultative nature of oligotrophy, as shown by the *K. pneumoniae *MB45, may contribute to the reported adaptation of remaining viable in hospital environment [28] for several days and cause nosocomial infection. Microbial contamination of working surfaces, clinical materials, and surgical devices poses a major threat in hospitals and intensive care units. With increasing threat because of greater incidence of multiple antibiotic resistant pathogens there is increased demand for novel disinfectants and disinfection methods. Due to exceptional physical and chemical properties of nanostructured materials there have been several attempts to improve the bactericidal activity of metal nanoparticles [31,67,68]. Most of these studies were confined to testing the nanoparticles on therapeutically sensitive test strains of *E. coli *(gram negative representative), *Bacillus subtilis *and *Staphylococcus aureus *(gram positive representatives). In an earlier study investigating the role of surface bound anionic species on zinc oxide quantum dots for the antibacterial activity against *E. coli*, it was shown that ZnO QDs having acetate ions had superior bactericidal activity than those described earlier [31]. In this study, the antibacterial potency of ZnO QDs was tested against the multiple antibiotic and serum resistant *K. pneumoniae *MB45. The bacterial growth rate was found to be inhibited with the increase in concentration of ZnO QDs under standard cultural conditions (Figure 3). Complete inhibition of growth of MB45 is observed at concentration of 500 mg/L ZnO QDs in the medium. Hence, in future ZnO QDs could be a good nanobiotic candidate for the control of multi-drug resistant pathogens as well as in disinfecting hospital environments, external wounds, medical and surgical devices; and also in suitable format may find application in drinking water treatment plants.

## Conclusion

The present work showed a new dihydrofolate reductase gene, *dfrA30 *responsible for high level of trimethoprim-resistance (>1500 mg/L and 1000 mg/L in *K. pneumoniae *MB45 and *E. coli *JM109 expressing *dfrA30 *gene cassette in pAK45 respectively). The strain MB45, isolated from river Mahananda was also resistant to nine more antibiotics as well as human serum; and was able to grow in very low nutrient condition for more than two days. Here we have suggested one of the possible ways to eradicate these pathogens by use of Zinc quantum dots (a nano-biotic) however other improved nano-biotics (experiments with ZnO-QDs having other capping agents are in progress) can also be used.

## Competing interests

The authors declare that they have no competing interests.

## Authors' contributions

All experimental work, data collection and analysis, phylogenetic analysis, literature search and writing of the draft manuscript were done by AK under the guidance of RC. ZnO- quantum dots were provided by SC and synthesis of ZnO- quantum dots was done by PJ. SC and PK were responsible for insilco mutational study of novel gene, *dfrA30*. RC was responsible for study concept, designing and coordinating the research, supervising the work and finalizing the manuscript. All authors have read, suggested changes/editing, and approved the final manuscript.

## Supplementary Material

Additional file 1 **Figure S1 - Sequence alignment between wild type (WT) and *Mycobacterium avium *Dfr (PDB ID: **2W3V**)**. Click here for file
