# Supplementary figures and images for: A multiple antibiotic and serum resistant oligotrophic strain, Klebsiella pneumoniae MB45 having novel dfrA30, is sensitive to ZnO QDs
Source: Ann Clin Microbiol Antimicrob. 2011 May 19;10:19. doi: 10.1186/1476-0711-10-19 (PMC3118321; doi:10.1186/1476-0711-10-19)

**
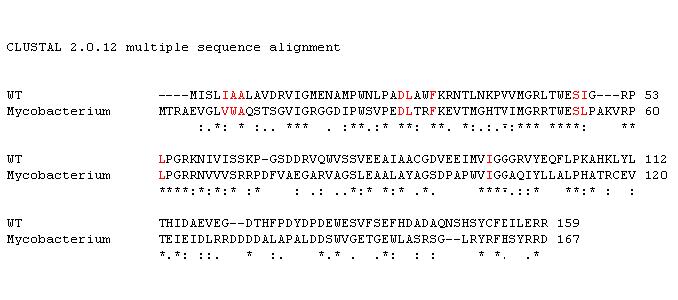
**

Supplement: Additional file 1 — Figure S1 - Sequence alignment between wild type (WT) and Mycobacterium avium Dfr (PDB ID: 2W3V). [file 1476-0711-10-19-S1.DOC]
